# Gut microbiota changes in preeclampsia, abnormal placental growth and healthy pregnant women

**DOI:** 10.1186/s12866-021-02327-7

**Published:** 2021-10-04

**Authors:** Lihui Huang, Min Cai, Li Li, Xin Zhang, Yang Xu, Jianhua Xiao, Qian Huang, Guijuan Luo, Zhaoyang Zeng, Cuiyuan Jin, Yuanxiang Jin, Jun He, Weitao Yang

**Affiliations:** 1 Changsha Hospital for Maternal and Child Health Care, Hunan, China; 2 Hengyang Medical College, University of South China, Hengyang, China; 3 College of Biotechnology and Bioengineering, Zhejiang University of Technology, Hangzhou, China

**Keywords:** Gut microbiota, Preeclampsia, Placental growth factor, Pregnant women, *Lactobacillus*

## Abstract

**Background:**

Preeclampsia (PE) is a condition of high blood pressure that is usually concurrent with proteinuria in pregnancy. PE complicates the management of both maternal and fetal health and contributes to most adverse pregnancy outcomes, but the mechanism underlying the development of PE remains unclear. In this study, we performed a case-control study to compare the gut microbiota of PE (*n* = 26), abnormal placental growth (APG, *n* = 25) and healthy pregnant women (*n* = 28) and analyzed the potential pathogenic role of gut microbiota in PE progression.

**Results:**

The clinical pathophysiological state did not affect the bacterial diversity, while the compositions of the gut microbiota were significantly altered in both the PE and APG groups compared with healthy pregnant women. At the phylum level, *TM7* was significantly increased in women with APG. Heterogeneity was observed at the genus level, especially in genera with positive LDA scores, suggesting the stage-dependent effect of gut microbiota on the development of PE. The beneficial bacterium *Lactobacillus* was markedly depleted in the PE and APG groups but was only correlated with blood pressure (BP) and proteinuria levels in the PE group. Two different bacterial taxa belonged to *Lactobacillus* showed different correlations (OTU255 and OTU784 were significantly related to PE and APG, respectively).

**Conclusions:**

Our results indicated that shifts in the gut microbiota might occur from the early stages of the development of PE, which is of possible etiological and therapeutic importance.

## Background

PE is a disorder of pregnancy characterized by the onset of hypertension and is usually concurrent with proteinuria in pregnancy [1]. Most PE occurs during the third trimester of pregnancy, and with generally worse outcomes when it occurs earlier [2, 3]. Since 2015, PE has become a leading cause of maternal and infant morbidity and mortality and affects 2 ~ 8% of all pregnancies worldwide annually [4, 5].

Although many studies have indicated that PE should be considered a subtype of metabolic disease [6, 7], the pathogenesis of PE has not been clarified, and there is no clear and effective clinical therapy. Some studies have suggested that placental obstruction, abnormal placentation, diffuse inflammatory response, vascular endothelial damage and environmental factors are possible etiologies of PE [1]. The most accepted pathogenesis of PE is placental dysplasia caused by anoxia, ischemia and hypoperfusion. In animal models, the increases in heterodimers formed by angiotensin II and bradykinin B2, two G protein-coupled receptors with opposing effects, can cause abnormal vasoconstriction, which triggers PE-like symptoms in pregnant mice [8]. However, the causes of the abovementioned abnormalities remain obscure; thus, pathogenetic heterogeneity complicates the management of both maternal and fetal health. For this reason, no reliable biomarkers or clinical tests can predict the emergence of PE in the earlier stages of pregnancy, and the only way to cure PE is termination [1].

A very encouraging and innovative hypothesis has recently been proposed whereby the mutual relationships between microbes and hosts could be an important factor affecting health [9]. Tens of trillions of microbes compose a complex ecosystem inhabiting mainly our intestine, also known as the gut microbiota [10], which has distinctive characteristics during pregnancy [11]. Numerous microbes in our gut help us digest complex and indigestible carbohydrates to produce beneficial metabolic output [12, 13]. Highly diverse bacterial-related proteins can maintain intestinal and immune homeostasis [14]. Dysbiosis in our gut is associated with metabolic disorders [15], immune system dysfunction [16], and even the development of obesity [17, 18] and diabetes [19, 20] of the host. However, the relationship between the gut microbiota and PE has remained largely unclarified until now. Some studies glimpsed the disturbance of both gut and placental microbiota in PE cohorts [21–23], but there has been a lack of stage-dependent and chronological analyses. In this study, healthy pregnant women and preeclamptic pregnant women were recruited. Because increasing evidence indicates that abnormal placentation is associated with PE [24] and that such abnormalities can often be predicted by clinical tests of placental growth factor (PIGF) [25] early in pregnancy, we also recruited 25 pregnant women with significantly decreased PIGF levels. Using 16S rRNA gene sequencing on stool samples, we revealed the gut microbiota profile of women with PE.

## Results

### Subject clinical features description

A total of 100 women were recruited, which included 21 healthy women (NW group), 28 healthy pregnant women (NP group), 25 pregnant women with abnormal placental growth (APG group, decreased PIGF Multiple of the Median, MoM, value) and 26 pregnant women with pre-eclampsia (PE group). There were no significant differences in age or body mass index (BMI) between the four groups. The gestational age of the APG group was significantly lower than those of the NP and PE groups. The setting of the APG group was more conducive to estimating gut microbiota changes at earlier gestational weeks, and the diastolic blood pressure (DBP), systolic blood pressure (SBP) and urinary protein concentration (UP) of the PE group were significantly higher than those of the other three groups (Table 1).

**Table 1:** Summary of subject characteristics

Groups	NW	NP	APG	PE
Subjects, n	21	28	25	26
Age range, years (SD)	31.04 ± 3.73	29.03 ± 3.80	27.50 ± 0.57	29.23 ± 4.85
gestational week (SD)	–	36.50 ± 7.43	23.71 ± 5.80*	36.73 ± 3.44
BMI (SD)	20.49 ± 1.30	21.87 ± 2.62	25.01 ± 3.55	24.27 ± 4.34
SBP, mmHg (SD)	110.95 ± 9.38	113.46 ± 8.79	115.00 ± 9.37	145.61 ± 10.44**
DBP, mmHg (SD)	71.38 ± 5.62	73.21 ± 7.28	70.27 ± 6.99	95 ± 7.14**
Urine protein, scores (SD)	0	0	0	1.57 ± 0.70**

All values are mean ± standard deviation. An asterisk indicated a significant difference (* at *p* < .05, ** *p* < .01) between the NP and the labelled group. Paired t test followed by FDR correction

### Effects of PE on the overall structure of the gut microbiota

To better analyze the reads, which ranged from 74,949 to 201,741 (approximately 124,761 clean reads per sample) obtained by fecal sample sequencing.

**Fig. 1:**
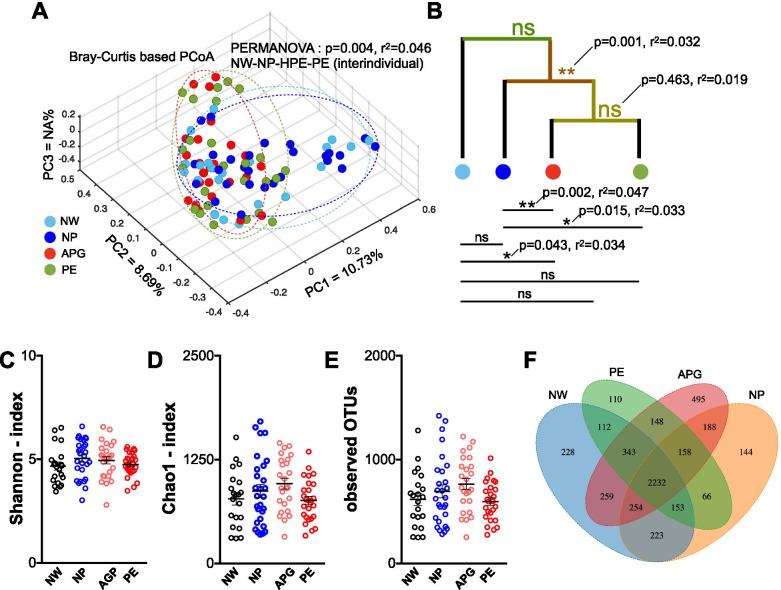
Effects of PE on the Overall Structure of Gut Microbiota. (**A**, **B**) Principal coordinate analysis based on Bray-Curtis distance of the bacterial communities in NW, NP, APG and PE. The differences in beta diversity between each paired groups were tested by permutational multivariate analysis of variance (PERMANOVA, FDR was controlled at 5%). (**C**, **D**, **E**) The Shannon, Chao1 and observed OTUs estimates of fecal microbiota in each group. (**F**) Venn diagram of the numbers of the identified OTUs. Dots in box plot show values in each individual. An asterisk indicated a significant difference (* at *p* < .05, ** *p* < .01) between the labelled groups

Compared with the NW group, principal component analysis (PCoA) based on the Bray-Curtis distance combined with PERMANOVA (also known as Adonis) test showed a significant difference (*p* = 0.043) in bacterial composition only to that of the APG group (Fig. 1A, B). As in the three groups of pregnant women, the gut microbiota compositions of the APG group, the PE group or all two groups of abnormal pregnancies (APG group plus PE group) were significantly shifted compared with that of the NP group (Adonis, *p* = 0.002, *p* = 0.015, *p* = 0.001, respectively) (Fig. 1B). Additionally, the PE group was significantly different from the NP group. The NW group showed no significant differences from the other groups when all the abnormally pregnant women were considered as a whole group. Although the pathophysiological state of pregnant women affected their gut microbiota, the alpha diversity, which included the Shannon and Chao1 indices, exhibited no statistical differences (Fig. 1C, D). The total number of OTUs did not differ significantly between any of the groups (Fig. 1E). For this analysis, only OTUs that are shared between 2 or more individuals within the group, were taken into account. A total of 2332 OTUs were observed in all four groups as the core shared features/OTUs (Fig. 1F), and the APG group harbored the highest number (495) of detectable unique OTUs.

### Gut microbiota compositional shifts in APG and PE at the phylum and genus level

**Fig. 2:**
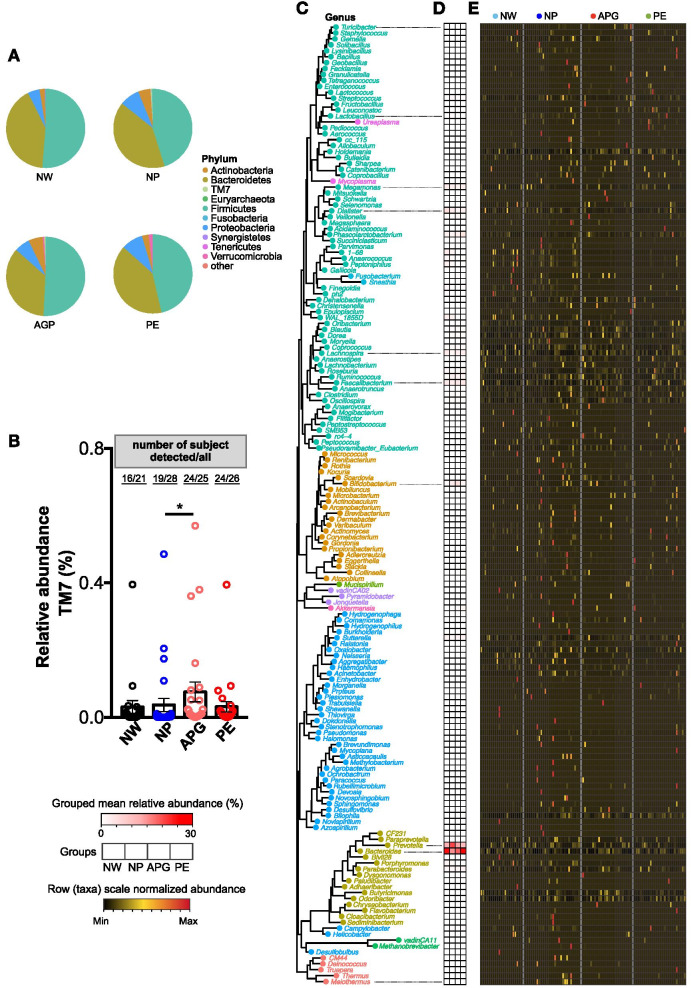
Gut microbiota compositional shifts in APG and PE at phylum and genus level. (**A**) Relative abundances of phyla in all the four groups. (**B**) Relative abundances of the phyla *TM7* in samples. An asterisk indicated a significant difference (* at *p* < .05, ** *p* < .01) between the labelled paired groups. Paired t test followed by FDR correction. (**C**) Neighbour joining phylogenetic tree of all the 162 annotatable genera. Text indicated the generic name of each bacterial taxa (plot) and the colour of the text and plot represented the level of phylum of each genus. (**D**) Mean relative abundance of all genera in each group. (**E**) Normalized abundance of each genus in samples

As many other studies have indicated, the main microbial communities in the human gut microbiota mainly belong to *Firmicutes*, *Bacteroidetes*, *Proteobacteria*, *Actinobacteria* and *Verrucomicrobia* [26]. The same results were found in our data (Fig. 2A). At phylum level, only the percentage of *TM7* was significantly increased in the APG group (Fig. 2B). Number of genera belonged to *Firmicutes* (68) was higher than that of *Proteobacteria* (40), *Actinobacteria* (22) and *Bacteroidetes* (16) (Fig. 2C). The most prevalent genera (at relative abundance level) detected in fecal samples were *g_Prevotella*, *g_Bacteroides*, *g_Faecalibacterium*, *g_Lachnospira*, *g_Megamonas* and *g_Dialister* across all subjects (Fig. 2D and E). Noteworthily, genera belonged to *Proteobacteria* and other rare phylum were more likely to be detected in NP subjects (Fig. 2C and E).

### Disturbance of gut microbiota was associated with the clinical characteristics of PE

**Fig. 3:**
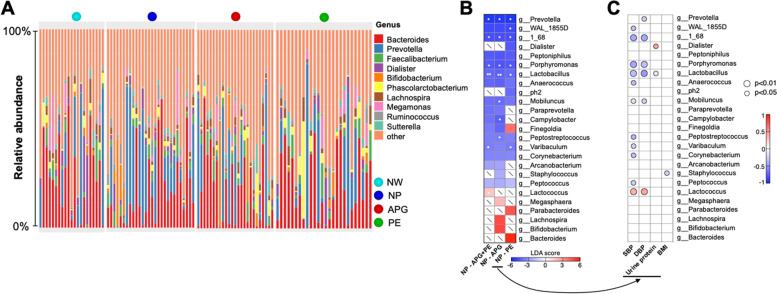
Disturbance of gut microbiota was associated with the development of PE. (**A**) Relative abundances of the most abundant ten genera in samples. (**B**) linear discriminant analysis effect size identified the most differentially abundant taxa between the selected two groups. The enriched taxa were indicated with a positive LDA score, and taxa enriched in NP have a negative score. Only taxa meeting an LDA significant threshold of > 3 were shown. The asterisk indicated a significant relative abundance change of the taxa (* at *p* < .05, ** *p* < .01) between the selected paired groups. Paired t test followed by FDR correction. (**C**) Correlations between the relative abundance of the selected genus and the clinical parameters

The composition of the gut microbiome at the genus level revealed by OTUs showed some more detailed changes in microbes. The compositions of the 10 most abundant genera in all subjects are shown in Fig. 3A. Apparently, *g_Prevotella* was the most common (detected in 19 subjects out of the 28 NP subjects) and prevalent (*p* = 0.017) genus in the NP group when compared with the NW group or any patient groups (Fig. 3A). To further identify the relationship between gut microbiota changes and the development of PE, linear discriminant analysis (LDA) was used to identify the core bacterial differences and revealed a total of ten genera with significant differences.

Specifically, the relative abundances of *g_Prevotella*, *g_WAL_1855D*, *g_1_68*, *g_Porphyromonas*, *g_Varibaculum* and *g_Lactobacillus* were significantly decreased in the PE group compared with the NP group (Fig. 3B). Between the APG and NP groups, *g_Prevotella*, *g_1_68*, *g_Porphyromonas*, *g_Lactobacillus*, *g_Mobiluncus*, *g_Campylobacter* and *g_Peptostreptococcus* were decreased significantly in the APG group (Fig. 3B). When considering the abnormally pregnant women as a whole group, 6 genera with significant differences were detected, whereby the significantly decreased abundance of *g_Prevotella*, *g_1_68*, *g_Porphyromonas*, *g_Lactobacillus*, and *g_Varibaculum* and the significantly increased abundance of *g_Lactococcus* (Fig. 3B) were compared to those of the NP group.

Obviously, *g_Lactobacillus* showed unanimous correlations with both blood pressure and UP (Fig. 3C). *g_1_68*, *g_Porphyromonas*, *g_Mobiluncus*, and *g_Lactococcus* were significantly correlated with SBP and DBP. Although there was no significant LDA difference in the PE group compared with the NP group (Fig. 3B), the relative abundance of *g_Staphylococcus*, a potential pathogen, was significantly (*p* = 0.029) inversely correlated with the BMI index in the NP and PE groups (Fig. 3C).

### The loss of *g_Lactobacilli* is only related to the abnormal clinical indicators of pre-eclampsia patients

**Fig. 4:**
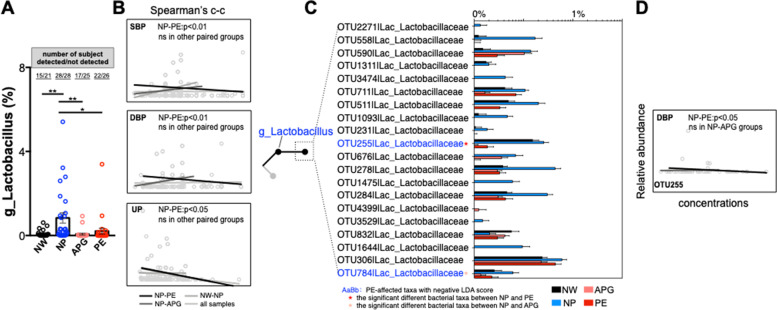
The loss of *g_Lactobacilli* is only related to the abnormal clinical indicators of Pre-eclampsia patients. (**A**) Relative abundances of *g_Lactobacilli* in samples. (**B**) The correlations between the relative abundance of *g_Lactobacilli* and the concentrations of SBP, DBP and UP. (**C**) The relative abundances of the OTUs belonged to the *g_Lactobacilli*. (**D**) The correlations between the relative abundance of OTU255 and the concentrations of DBP

Although the abundance of g_*Lactobacillus* in human gut is very low, it has been regarded as the most common probiotic in keeping human health and has been used worldwide in food processing, drug development and clinical treatment over the past decades [27]. In our cohort, *g_Lactobacillus* was one of the most detectable genus in the four groups, rates of number of subject detected g_*Lactobacillus* /all ranged from 68 to 100% (Fig. 4A). The ratio of number of subject detected g_*Lactobacillus* /all and the relative abundance of *g_Lactobacillus* were significantly higher in the NP group than in any other group (Fig. 4A), implying that *g_Lactobacillus* may correlate with the health of pregnant women. Moreover, we analyzed the correlations between the relative abundance of *g_Lactobacillus* and blood pressure and UP in different grouping modes. The abundance of *g_Lactobacillus* was significantly (*p* = 0.0042, 0.0029 and 0.031, respectively) inversely correlated with SBP, DBP and UP in the NP and PE groups (Fig. 4B). However, in the NW and NP, NP and APG or all sample groups, no statistically significant correlations were found (Fig. 4B). We further found that 20 OTUs (bacterial taxa) belonged to *g_Lactobacillus,* and 2 OTUs, OTU255 and OTU784, showing significant differences (*p* = 0.017 and 0.023, respectively, and both of them had negative LDA scores) (Fig. 4C). The relative abundance of OTU255 was significantly decreased in the PE group compared with the NP group, and OTU784 was decreased with significance only between the APG and NP groups (Fig. 4C). Only OTU255 was significantly negatively related to DBP in the PE and NP groups (Fig. 4D). These results reveal profound changes in the intestinal microbiome structure of the APG and PE groups, indicating the importance of gut microbiota changes in the development of PE.

## Discussion

Pregnancy is one of the most special and vulnerable physiological states of humans under natural conditions. The maintenance of physical and mental health during pregnancy means a great deal to both maternal and fetal safety. Since PE was first described by Hippocrates [24], toxemia caused by external toxic substances, nutritional imbalance, genetic factors and placental dysfunction have been proposed as the pathogenesis of PE. The heterogeneity of etiology led to the lack of effective methods for both clinical prediction, prevention and intervention. We aimed to explore the potential role of the gut microbiota in the development of PE. To our knowledge, the cohort in this study was the first to include pregnant women with abnormal PIGF levels. Our results proposed that gut microbiota dysbiosis could play an important role in the development of PE in a chronological manner.

Microbial community disturbances in the gut [21–23] and the placenta [28] were previously detected in PE members, and using fecal microbiota transplantation, the gut–placenta axis was proposed [23]. Consistent with previous studies [23], we observed that the composition of the gut microbiota in the PE group was clearly and firmly shifted (Fig. 1A, B). Exclusively in our data, a more robust shift of the gut microbiota was also detected in the APG group (Fig. 1A, B). If we took all the abnormally pregnant women as a whole group, the gut microbiota was also significantly different from that of healthy women (Fig. 1B). Gestational age was one of the main factors affecting pregnant women’s gut microbiota [11, 21]. Interestingly, there was no difference between the PE and APG groups (Adonis, *p* = 0.463), even though a notable gestational age gap existed (Table 1), indicating that gut microbiota changes may have occurred in the early stage of pregnancy (considering the abnormal PIGF levels implied the abnormal pathophysiology of the pregnant women). Many diseases, such as obesity [17, 18], colorectal cancer [29] and arteriosclerosis [30], are accompanied by decreased gut microbiota diversity. A relatively high diversity of gut microbiota is considered a sign of health [31]. However, no significant differences in the Shannon and Chao1 indexes and the observed OTUs were observed (Fig. 1C, D).

The relative abundance of *TM7* was significantly increased in the APG (Fig. 2B). The most prevalent genera were mainly belonged to *Firmicutes* and *Bacteroidetes*. Bacteria belonged to *Firmicutes* are believed to help us to improve the utilization of calories in food [32], and more prevalent in all the pregnant women group (NP, APG and PE) (Fig. 2E). However, more bacteria belonging to *Bacteroidetes*, which usually contain more gram-negative bacteria that can produce lipopolysaccharide [33, 34], were observed in the APG and PE (Fig. 2D, E).

More significant were the patterns of gut dysbiosis in the APG and PE. The relative abundance of *g_Prevotella* was significantly (*p* = 0.033 and 0.019, respectively) decreased (from ~ 20% in NP down to ~ 10% in APG and PE with significance). *g_Prevotella* is a main functional bacterial group in the human intestinal tract [35]. Recent studies have reported that *g_Prevotella* can use fiber and polysaccharides to produce short-chain fatty acids [36], such as butyrate, the main energy source of intestinal epithelial cells [37] and the regulator of colonic T cell differentiation [38]. At the same time, the colonization of *g_Prevotella* is also helpful to resist infections with pathogens [39, 40]. The beneficial effects of probiotics on human health have been widely studied. For pregnant women, the role of probiotics has not been fully revealed. *g_Lactobacillus* is the dominant bacteria in the female vagina for its viability in low pH and glycose condition [41] and has been considered a probiotic in keeping human health, which is associated with numerous beneficial outcomes [42, 43]. Although *g_Lactobacillus* is not the predominance genus in either male or female individuals gut [44], it is detectable in the maternity gut microbiota [21, 45]. In our cohort, we found that the *g_Lactobacillus* was detected, unexpectedly, in all of the NP subjects (Fig. 4A). Its relative abundance was significantly higher than that of any of the other three groups, implying that *g_Lactobacillus* may play an active role in healthy pregnancy (Fig. 4A). The abundance of *g_Lactobacillus* was significantly inversely related to SBP, DBP and the concentration of urine protein. Such microbiota shifts may be the result of physiological changes (such as hormone homeostasis) in the female body during pregnancy. For example, progesterone can be used as a nutrient to help the colonization and proliferation of *Bifidobacteria* in pregnant women’s feces [46]. A study of 33,399 primiparas showed that daily or weekly intake of *Lactobacillus* or their yogurt products during pregnancy significantly reduced the risk of PE [47]. Another study of 70,149 Norwegian mothers showed a significant correlation between supplementation of probiotic milk in late pregnancy and a lower risk of PE [48]. The loss of beneficial and functional genera may lead to the breeding of potential pathogens in both APG and PE, such as *g_Lactococcus*, and ultimately contribute to the development of PE. Correlations between clinical features and gut microbiota features provide potential evidence for future microbiota-targeted therapy, such as the reduction of the relative abundance of the SBP and (or) DBP positively related bacteria or treating potential probiotics (microbe inversely related to SBP and DBP) and (or) its corresponding prebiotics. In the future, probiotic supplementation and gut microbiota-targeted special diets may have therapeutic effects in improving or blocking the occurrence and development of preeclampsia and should be explored.

## Conclusions

Taken together, we clearly revealed gut microbiota dysbiosis in PE and APG. This shift in the gut microbiota may occur from the early stages of the development of PE, and future longitudinal multiregion large-cohort studies are needed to constitute a microecological perspective for PE management.

## Materials and methods

### Study subject recruitment

All subjects were from the outpatient department or ward of Changsha Hospital for Maternal and Child Health Care, Hunan, China. The definition of PE was according to the diagnosis standards, and the subjects were screened by experienced clinicians. The diagnostic standards were as follows: hypertension (systolic blood pressure ≥ 140 mmHg and/or diastolic blood pressure ≥ 90 mmHg) and high urine protein (≥ 0.3 g/24 h urine collection or random dipstick reading urine protein positive). Additionally, PE women with systolic blood pressure ≥ 160 mmHg and/or diastolic blood pressure ≥ 110 mmHg, thrombocytopenia, impaired liver/kidney function, pulmonary edema or emerging nervous system abnormalities were clinically defined as severe. For the definition of patients with abnormal PIGF values in early and middle pregnancy, the values were obtained by normalizing the concentrations of PIGF with the median value in the localized database (anthropometry-matched pregnant women’s database), also known as the MoM value, in early and middle pregnancy. Patients with malignant tumors, depression, cardiovascular diseases, diabetes, chronic nephritis, liver and kidney function damage or immune system diseases were excluded.

### Fecal sample collection and DNA extraction

Fecal samples of each subject were obtained at home. Fresh feces were immediately removed into our storage kit and transferred to a 4 °C domestic refrigerator. Within 1 day, the stool samples were sent to our outpatient clinic (4 °C) and stored at − 80 °C until further processing. The reagent for fecal collection and storage made it possible to deposit stools at ambient temperature for up to a month with minimal alterations when compared with deeply frozen samples (Zhejiang Hangzhou Equipment Preparation NO: 20190682, GUHE Laboratories, Hangzhou, China). Stool samples was excluded if any organic change is detected.

### 16S rRNA gene amplicon sequencing and analysis

Microbial genomic DNA was extracted from frozen fecal pellets using the e.Z.N.A. TM stool DNA kit (OMEGA Bio-Tek, USA) following the manufacturer’s instructions. The resultant DNA was quantified by Nanodrop and then stored at − 80 °C for further analysis. The V4 region of the 16S rRNA gene was PCR-amplified from microbial genomic DNA using primers (forward primer, 5′-GTG CCA GCM GCC GCG GTA A-3′; reverse primer, 5′-GGA CTA CHV GGG TWT CTA AT-3′). The PCR products were detected using dual-indexing amplification and sequencing approaches on the Illumina MiSeq platform. Briefly, raw sequences equipped with barcodes were assigned to their unique corresponding samples and identified as valid sequences. The following criteria were used for inferior sequence filtering: (i) sequences with a < 150-bp length or < 20 average Phred score and (ii) sequences that contained ambiguous bases or > 8-bp mononucleotide repeats, and the average number of clean reads from each sample was 124,761. Qualified paired-end reads were matched (blasted with each other), dereplicated (−-derep_fulllength), clustered (−-cluster_unoise) and chimeras filtered (−-uchime3_denovo) using VSEARCH (V2.4.4) against the SILVA138 database and then assembled into operational taxonomic units (OTUs) with sequence similarity ≥97% using the Quantitative Insights Into Microbial Ecology (QIIME2, v2020.6) pipeline. The OTUs containing less than 0.001% of total sequences across all samples were discarded. To minimize the difference in sequencing depth across samples, an averaged, rounded rarefied OTU table was generated by averaging 100 evenly resampled subsets under 90% of the minimum sequencing depth for further analysis. Measurements of OTU-level alpha diversity, such as the Chao1, Richness, abundance-based coverage estimator (ACE), Shannon and Simpson indices of each sample, were calculated using the OTU table in QIIME2. Maximum likelihood phylogenetic tree was constructed with phangorn (v.2.5.5) using a neighbour joining tree as the starting point.

### Statistical analysis

Differences between two or more groups were evaluated by one-way ANOVA followed by Dunnett’s test or Fisher’s protected least significant difference test using SPSS 24.0 (SPSS, Chicago, Illinois). Values are expressed as the mean ± SEM. The significance of the differentiation of microbiota structure among groups was assessed by permutational multivariate analysis of variance (PERMANOVA) using the R package “vegan”.

## Data Availability

The datasets used and/or analysed during the current study are available from the corresponding author on reasonable request. All 16S rRNA raw data have been submitted to China National GeneBank DataBase, CNGBdb (accession number, CNP0001512).

## Abbreviations

PE: Pre-eclampsia
APG: Abnormal placental growth
BP: Blood pressure
PIGF: Placental growth factor
BMI: Body mass index
DBP: Diastolic blood pressure
SBP: Systolic blood pressure
UP: Urinary protein
OTUs: Operational taxonomic units
PCoA: Principal component analysis
F/B: Firmicutes and Bacteroidetes
LDA: Linear discriminant analysis
LPS: Lipopolysaccharide
SCFAs: Short chain fatty acids
